# Synchronous Cervical and Vulvar High-Grade Squamous Intraepithelial Lesions with Unusual p16/p53 Immunophenotype: A Case Report

**DOI:** 10.3390/reports9020118

**Published:** 2026-04-11

**Authors:** Catalin-Bogdan Satala, Alina-Mihaela Gurau, Andrei-Ionut Patrichi, Andreea Onofrei (Popa), Daniela Mihalache

**Affiliations:** 1 Faculty of Medicine and Pharmacy, Medical and Pharmaceutical Research Center, “Dunărea de Jos” University of Galati, 800008 Galati, Romania or stlcatalin92@yahoo.com (C.-B.S.); andreea.onofrei@ugal.ro (A.O.); daniela.mihalache@ugal.ro (D.M.); 2 Department of Pathology, Clinical County Emergency Hospital Braila, 810325 Braila, Romania; 3 The School for Doctoral Studies in Biomedical Sciences, “Dunărea de Jos” University of Galati, 800008 Galati, Romania; 4 The Doctoral School of Medicine and Pharmacy, “George Emil Palade” University of Medicine, Pharmacy, Science and Technology, 540142 Targu Mures, Romania; andrei-ionut.patrichi@umfst.ro

**Keywords:** p16, p53, cervical HSIL, vulvar HSIL, synchronous intraepithelial lesion, discordant immunophenotype

## Abstract

**Background and Objectives**: Synchronous cervical and vulvar squamous intraepithelial lesions are rarely reported. In most cases, these lesions are associated with high-risk human papillomavirus (HPV) infection and follow the conventional HPV-related pathway. Rarely, vulvar lesions may show an unusual immunohistochemical profile, with block-type p16 expression accompanied by aberrant p53 staining, creating diagnostic and etiopathogenetic challenges. **Case Presentation**: We report the case of an 83-year-old woman who presented with metrorrhagia and a symptomatic vulvar lesion. Histopathological evaluation revealed synchronous high-grade squamous intraepithelial lesion of the cervix and vulvar high-grade squamous intraepithelial lesion (VIN 3). Immunohistochemically, the cervical lesion showed block-type p16 positivity and a wild-type p53 pattern, supporting a conventional HPV-associated profile. In contrast, the vulvar lesion also demonstrated block-type p16 positivity, but with aberrant p53 overexpression, representing an unusual double-positive immunophenotype. **Conclusions**: This case highlights a rare presentation of synchronous lower genital tract squamous intraepithelial lesions with divergent immunophenotypic features. Accurate interpretation requires integration of morphology and immunohistochemistry, while the absence of direct HPV testing and *TP53* molecular analysis limits definitive etiopathogenetic classification. Reporting such cases may broaden awareness of unusual vulvar precursor lesions and potential diagnostic pitfalls in routine practice.

## 1. Introduction and Clinical Significance

High-grade squamous intraepithelial lesion (HSIL) of the cervix, corresponding histologically to cervical intraepithelial neoplasia grades 2 and 3 (CIN 2 and CIN 3), is the principal preinvasive squamous precursor of cervical squamous cell carcinoma. These lesions are strongly associated with persistent infection by high-risk human papillomavirus (HPV) and occur predominantly in younger women, reflecting the epidemiology of HPV-related cervical disease. Their importance is emphasized by the continuing global burden of cervical cancer, which remains the fourth most common cancer in women worldwide [1,2].

In the vulva, squamous precursor lesions are currently divided into two biologically distinct entities: vulvar high-grade squamous intraepithelial lesion (vulvar HSIL), which is HPV-associated, and differentiated vulvar intraepithelial neoplasia (dVIN), which is HPV-independent. Vulvar HSIL corresponds to the former usual-type vulvar intraepithelial neoplasia and includes previously designated VIN 2 and VIN 3. This lesion is more often encountered in younger women and is linked to high-grade HPV infection. By contrast, dVIN usually arises in older women, often associated with chronic dermatoses, and is clinically important because of its stronger association with progression to invasive squamous cell carcinoma [1,3].

Synchronous squamous intraepithelial lesions involving the cervix and vulva are uncommon. When they occur, they are generally interpreted in the context of multicentric lower genital tract disease, most often related to persistent HPV infection [4,5]. However, rare cases may show discordant immunohistochemical profiles, particularly at the vulvar site, where block-type p16 expression may coexist with aberrant p53 staining [6].

In the vulva, interpretation may become particularly challenging when morphology and immunohistochemistry do not align with the expected binary model of HPV-associated versus HPV-independent precursor disease. In routine practice, block-type p16 expression generally supports vulvar HSIL, whereas aberrant p53 staining raises concern for an HPV-independent pathway, particularly differentiated vulvar intraepithelial neoplasia (dVIN). However, this distinction is not absolute. Rare vulvar squamous precursor lesions may show a discordant immunophenotype, including block-type p16 positivity together with aberrant p53 expression, creating a potential diagnostic pitfall. In such cases, p16 and p53 results should not be interpreted in isolation, but rather integrated with the morphologic features and, where available, molecular HPV and *TP53* testing [6,7,8].

The main purpose of presenting this case is not only to document a rare synchronous cervical and vulvar squamous intraepithelial presentation, but also to highlight a practical diagnostic challenge. In routine gynecologic pathology, vulvar squamous precursor lesions are often interpreted within a simplified HPV-associated versus HPV-independent framework. However, unusual combinations of morphology and immunohistochemistry, such as block-type p16 expression together with aberrant p53 staining, may create uncertainty regarding classification, with potential implications for interpretation, clinical communication, and management. This case is therefore presented as a diagnostic teaching example emphasizing the need for integrated morphologic, immunohistochemical, and, where available, molecular assessment.

## 2. Case Presentation

### 2.1. Presentation and Clinical Evaluation

An 83-year-old woman was referred to the Department of Gynecology, Brăila County Emergency Clinical Hospital, in January 2026 for evaluation of postmenopausal uterine bleeding that has persisted for approximately three months. In addition, she reported a lesion involving the left hemivulva, which had been present for more than one year and had recently become symptomatic, with vulvar pruritus and burning sensation, raising clinical concern because of local discomfort and apparent progression. According to the available history, the patient was an active smoker, was not vaccinated against HPV and had never undergone cervical cancer screening. Her past medical history was otherwise poorly documented. The only recorded comorbidities and prior surgical events were chronic venous insufficiency, cholecystectomy performed in 2017 and a ventral hernia without obstruction, diagnosed in 2018. No reliable information could be obtained regarding previous HPV-related lesions, prior gynecologic dysplasia, or immunosuppressive conditions.

Colposcopic examination revealed a whitish, well-demarcated peri-orificial area of the cervix measuring 0.8 × 0.6 cm. On the left hemivulva, a flat whitish lesion measuring 1.4 × 0.5 cm was identified. Biopsy sampling from both lesions was recommended for histopathological evaluation.

### 2.2. Histopathological Assessment

The cervical biopsy consisted of multiple fragments of squamous epithelium, most without attached underlying stroma, with associated hemorrhagic material. The epithelium showed full-thickness loss of maturation, marked nuclear pleomorphism and hyperchromasia, increased mitotic activity involving all epithelial layers, and scattered atypical mitotic features. These features supported the diagnosis of a high-grade squamous intraepithelial lesion/cervical intraepithelial neoplasia (HSIL/CIN3). No invasive component was identified in any of the examined fragments.

**Figure 1:**
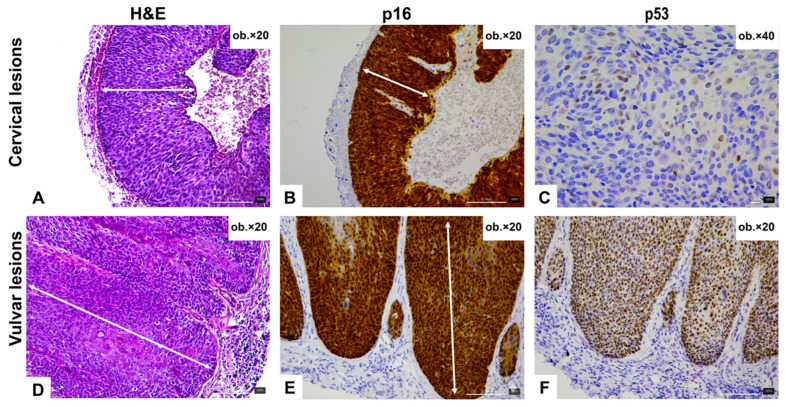
Representative histological and immunohistochemical findings in the cervical and vulvar squamous intraepithelial lesions. (**A**–**C**) Cervical high-grade squamous intraepithelial lesion showing complete loss of epithelial maturation on H&E (**A**), diffuse block-type p16 expression (**B**), and a wild-type p53 staining pattern with variable nuclear intensity (**C**). (**D**–**F**) Vulvar squamous intraepithelial lesion showing full-thickness atypia and impaired maturation on H&E (**D**), diffuse block-type p16 expression (**E**), and aberrant p53 overexpression with strong and diffuse nuclear staining (**F**). Arrows indicate the most diagnostically relevant epithelial areas.

The vulvar biopsy comprised fragments of squamous epithelium with underlying fibroconnective stroma. The lesional epithelium showed marked architectural disorganization with loss of maturation throughout the epithelial thickness. Prominent cytologic atypia was present, including enlarged hyperchromatic nuclei, irregular nuclear contours, increased nuclear-to-cytoplasmic ratio, and mitotic figures extending into the upper epithelial layers. Although the basal and parabasal layers displayed conspicuous atypia, the atypia was not confined predominantly to the basal compartment, but instead involved the full thickness of the epithelium. Importantly, we did not identify the morphologic features more typically associated with differentiated vulvar intraepithelial neoplasia (dVIN), such as premature maturation, prominent keratinization, dyskeratosis, or atypia largely limited to the basal layer. In the examined biopsy material, no associated dermatosis was identified histologically. Koilocytic change was not prominent. Taken together, the overall morphologic findings were considered more consistent with vulvar high-grade squamous intraepithelial lesion (HSIL), corresponding to vulvar intraepithelial neoplasia grade 3 (VIN 3), rather than dVIN. No stromal invasion was identified in the examined fragments. Representative histopathologic images are shown in Figure 1.

### 2.3. Immunohistochemical Findings

Immunohistochemical studies were performed on both biopsy specimens to further characterize the intraepithelial squamous lesions and support etiologic interpretation. In the cervical biopsy, p16 showed block-type positivity, supporting an HPV-associated phenotype, while p53 displayed a wild-type staining pattern, with variable nuclear staining intensity (Figure 1). In the vulvar biopsy, p16 likewise demonstrated block-type positivity within the lesional squamous epithelium. In contrast, p53 showed an aberrant overexpression-type pattern, characterized by strong and diffuse nuclear staining (Figure 1). In view of the unusual combination of block-type p16 expression and aberrant p53 staining, the immunophenotype was interpreted cautiously and in conjunction with the lesion morphology, which was favored to represent vulvar HSIL.

### 2.4. Clinical Outcome and Follow-Up

The immediate post-procedural course was uneventful. Following histopathologic confirmation of synchronous high-grade squamous intraepithelial lesions involving the cervix and vulva, the patient was referred to a tertiary care center with specialized gynecologic oncology services for further diagnostic evaluation, staging, and therapeutic planning within a multidisciplinary setting. At the time of manuscript preparation, no additional clinical records regarding excision, final staging, definitive treatment, or short-term outcome were available to the authors.

## 3. Discussion

The present case illustrates an unusual constellation of findings involving synchronous high-grade intraepithelial lesions of the cervix and vulva in an elderly patient, accompanied by divergent immunophenotypic profiles, with vulvar lesion demonstrating a mixed p16/p53 staining pattern. While HPV-associated and HPV-independent pathways of squamous carcinogenesis in the lower genital tract are generally considered distinct entities, increasing molecular evidence suggests that the biological landscape of these lesions may be more heterogeneous than previously appreciated [8].

### 3.1. Synchronous Intraepithelial Lesions of the Lower Genital Tract

The occurrence of synchronous intraepithelial lesions affecting multiple sites of the lower genital tract has been well documented in association with persistent oncogenic HPV infection. The concept of a shared epithelial “field effect” has been proposed to explain the presence of multifocal dysplastic lesions across anatomically contiguous mucosal surfaces. In this model, exposure to common carcinogenic factors, particularly HPV infection, may predispose multiple epithelial compartments to neoplastic transformation [6].

Nevertheless, the clinical context of the present case is somewhat atypical. Multifocal HPV-related disease is most frequently observed in younger patients, whereas vulvar precursor lesions identified in elderly individuals more commonly arise through HPV-independent mechanisms. An important differential diagnostic consideration in the present case was differentiated vulvar intraepithelial neoplasia (dVIN), particularly given the patient’s advanced age and the aberrant p53 staining pattern observed in the vulvar lesion. This possibility was carefully considered. However, the lesion showed full-thickness loss of maturation, diffuse cytologic atypia extending well beyond the basal layer, and mitotic activity in the upper epithelial layers—features that favor vulvar HSIL. By contrast, morphologic features more typical of dVIN, including premature maturation, prominent keratinization, dyskeratosis, and atypia predominantly restricted to the basal/parabasal compartment, were not identified in the examined material. No associated dermatosis was recognized histologically in the available biopsy. Therefore, despite the unusual p16/p53 immunophenotype, the overall morphology was considered more supportive of vulvar HSIL than dVIN. This distinction is diagnostically important because lesions with discordant morphology and immunohistochemistry may be misclassified if interpretation relies excessively on a single biomarker. In such cases, morphology should remain the primary basis for classification, while p16 and p53 are best interpreted as supportive rather than standalone determinants.

**Figure 2:**
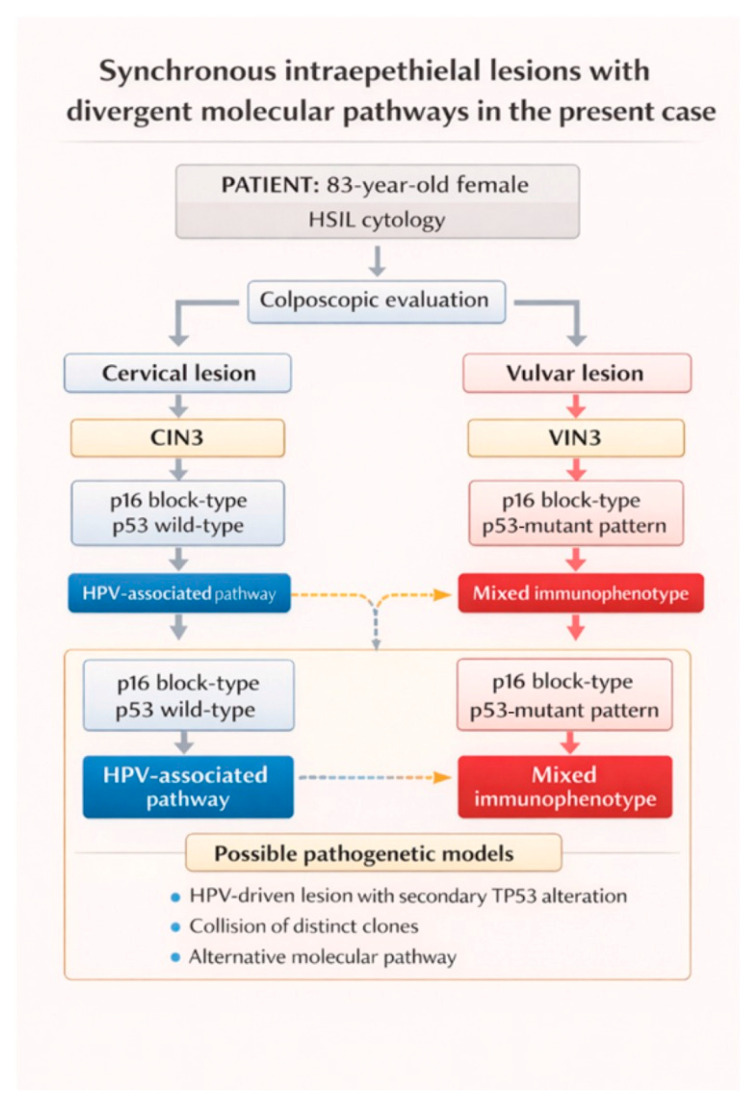
Diagnostic workflow and immunophenotypic profile of the synchronous cervical and vulvar lesions in the present case. Schematic representation of the diagnostic pathway in an 83-year-old patient with HSIL. Colposcopy revealed both a cervical and a vulvar lesion. Histopathological examination showed CIN3 and VIN3. Immunohistochemistry demonstrated block-type p16 expression with wild-type p53 in the cervical lesion, consistent with an HPV-associated pathway, whereas the vulvar lesion showed block-type p16 expression together with mutant-type p53 staining, indicating a mixed immunophenotype.

The coexistence of a cervical lesion demonstrating a typical HPV-associated immunophenotype together with a vulvar lesion showing a distinct molecular profile therefore raises the possibility that independent carcinogenic processes may develop concurrently within different epithelial compartments of the lower genital tract, as summarized in Figure 2.

Genomic studies have increasingly highlighted the complexity of carcinogenesis in vulvar squamous lesions. For example, Nooij et al. performed genomic sequencing of vulvar squamous cell carcinomas and precursor lesions and identified distinct molecular subgroups defined by HPV status and *TP53* mutational profile. Their analysis showed recurrent *TP53* mutations in 42% of HPV-negative precursor lesions and 68% of HPV-negative carcinomas, while *NOTCH1* mutations were found in 28% and 41%, and *HRAS* mutations in 20% and 31%, respectively. Importantly, a substantial subset of HPV-negative precursor lesions (35/60, 58.3%) and carcinomas (10/29, 34.5%) remained *TP53* wild-type, suggesting that vulvar squamous neoplasia cannot always be explained by a strictly dichotomous model. These findings support the existence of additional molecular heterogeneity within vulvar squamous precursor lesions and may help explain unusual or discordant immunophenotypes [9].

### 3.2. Overlapping Immunophenotypes in Vulvar Squamous Lesions

One of the most notable findings in the present case is the coexistence of p16 overexpression and mutation-type p53 staining in the vulvar lesion. Traditionally, these two markers have been interpreted as reflecting distinct pathogenetic mechanisms. HPV-associated lesions typically demonstrate diffuse p16 expression as a consequence of *RB* pathway inactivation mediated by HPV E7 oncoprotein, whereas HPV-independent lesions are frequently characterized by *TP53* mutations that result in abnormal p53 protein accumulation detectable by immunohistochemistry [10,11,12,13,14,15].

However, recent studies have shown that these molecular pathways may occasionally overlap. Tessier-Cloutier et al. performed molecular characterization of vulvar squamous neoplasia and demonstrated that although the majority of tumors could be categorized as either HPV-associated or *TP53*-driven, a subset of cases displayed overlapping molecular features, including tumors with both transcriptionally active HPV infection and *TP53* mutations. In their series, *TP53* mutations were identified in 22 of 30 vulvar squamous cell carcinomas (73%) and 11/13 dVIN lesions (85%), while mutational progression from in situ to invasive disease was observed in 7 of 26 matched cases (27%) and usually involved *TP53* alterations. Their findings suggest that additional genomic alterations may occur during tumor evolution, resulting in more complex molecular profiles than those predicted by the traditional model [16].

Similarly, Yang et al. evaluated the classification of vulvar squamous lesions using combined p16 and p53 immunohistochemistry and reported that while most lesions followed the expected staining patterns, diagnostically relevant caveats persisted, particularly in cases with non-complementary or “double-positive” p16/p53 profiles. In their series, five of 82 p16-positive VSCCs (6.1%) showed abnormal p53 staining despite block-type p16 expression; these cases were HPV ISH-negative and harbored *TP53* mutations, and were therefore classified as HPV-/p53 abnormal. They further showed that combined immunohistochemistry may refine, and in some cases revise, the classification of vulvar precursor lesions, highlighting the importance of integrated interpretation rather than reliance on a single biomarker [6].

This distinction is clinically relevant because different precursor lesions may lead to different therapeutic approaches and follow-up strategies. Vulvar HSIL is generally managed within the framework of HPV-associated lower genital tract disease, often with excision, ablation, or topical therapy depending on lesion extent and patient factors, whereas dVIN is typically approached more cautiously because of its stronger association with rapid progression to invasive squamous cell carcinoma and its frequent occurrence in a background of chronic vulvar dermatosis. For this reason, when a vulvar lesion shows discordant morphology and immunohistochemistry, diagnostic precision is important not only for classification but also for appropriate clinicopathologic communication and risk framing. To place the present case in a practical diagnostic context, the main morphologic, immunohistochemical, and clinical distinctions between conventional vulvar HSIL, differentiated VIN, and the current case are summarized in Table 1.

### 3.3. Possible Biological Mechanisms Underlying Mixed p16/p53 Patterns

Several biological mechanisms may potentially account for the coexistence of p16 overexpression and mutation-type p53 staining within the same lesion. Although the exact mechanism remains uncertain, three main hypotheses have been proposed in the literature: secondary *TP53* alterations occurring during HPV-driven carcinogenesis, collision phenomena involving independent epithelial clones, and alternative molecular pathways of tumor development [17,18,19].

*TP53* mutations may arise as secondary genetic events during the progression of an initially HPV-driven lesion. In this scenario, viral oncogene activity initiates neoplastic transformation, while subsequent genomic instability facilitates the acquisition of additional driver mutations, including alterations affecting *TP53* [17,18,19,20].

Evidence supporting this mechanism has been reported in several molecular studies of vulvar carcinoma. Tessier-Cloutier et al. identified *TP53* mutations in a subset of tumors demonstrating p16 overexpression, suggesting that HPV-associated tumors may acquire additional genetic alterations during clonal evolution. Similar observations have been reported in studies of genital squamous carcinomas in which *TP53* mutations were detected in tumors harboring transcriptionally active HPV infection [15,20].

These findings support the concept that HPV-associated carcinogenesis may represent a dynamic process in which secondary genomic events contribute to tumor progression and phenotypic diversification [20]. An alternative explanation involves the coexistence of distinct epithelial clones within the same lesion. In this scenario, separate neoplastic populations harboring different molecular alterations may arise independently and subsequently coexist within a single biopsy specimen [21].

Intratumoral heterogeneity has been increasingly recognized as a common feature of epithelial malignancies. Kortekaas et al. performed genomic profiling of vulvar squamous cell carcinoma and demonstrated considerable molecular heterogeneity, with multiple genetic alterations affecting different signaling pathways within the same tumor. These findings support the possibility that distinct clonal populations may coexist within vulvar squamous lesions [13].

Collision phenomena involving independent neoplastic clones have been described in a variety of epithelial tumors and may account for unusual or heterogeneous immunohistochemical profiles observed in routine diagnostic practice. In the context of the present case, the possibility that the mixed immunophenotype reflects the coexistence of separate HPV-associated and *TP53*-mutant epithelial clones cannot be excluded [22,23,24].

A third hypothesis proposes that certain vulvar squamous lesions may arise through alternative molecular pathways that remain incompletely characterized. While the traditional model of vulvar carcinogenesis distinguishes between HPV-associated and HPV-independent pathways, recent genomic studies have revealed a broader spectrum of genetic alterations involved in these tumors [6,8,9].

**Figure 3:**
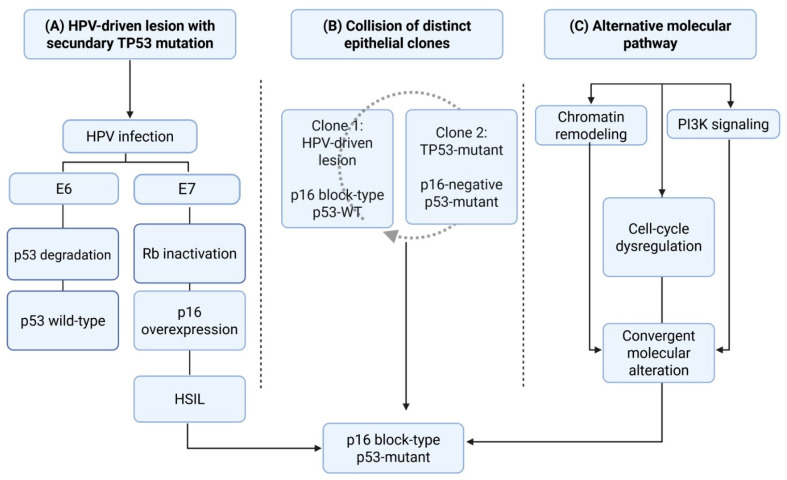
Proposed biological mechanisms underlying the mixed p16 block-type/p53-mutant in vulvar squamous lesions. (**A**) An initially HPV-driven lesion may acquire secondary *TP53* alterations during clonal evolution, resulting in retention of p16 overexpression together with a p53-mutant phenotype. (**B**) A mixed immunophenotype may reflect collision or clonal admixture between two distinct epithelial populations, namely an HPV-associated clone and a TP53-mutant clone lacking the conventional HPV-associated profile. (**C**) Some lesions may arise through alternative molecular pathways involving convergent genomic alterations, including abnormalities affecting chromatin remodeling, PI3K signaling, and cell-cycle regulation, ultimately producing an unusual combined immunophenotype. The final common outcome in all three scenarios is a vulvar squamous lesion showing block-type p16 expression together with abnormal p53 staining.

Large-scale sequencing studies have identified recurrent mutations affecting genes involved in chromatin remodeling, *PI3K* signaling, and cell-cycle regulation in vulvar squamous carcinomas. These findings suggest that the molecular landscape of vulvar neoplasia is more complex than previously appreciated and that some lesions displaying unusual immunophenotypic patterns may arise through combinations of molecular alterations that do not correspond strictly to the classical HPV-dependent or *TP53*-driven pathways [17,18,19]. Taken together, these three hypotheses are summarized schematically in Figure 3.

The coexistence of synchronous cervical and vulvar lesions in the present case raises at least two biologically plausible interpretative models. One possibility is a field effect or field cancerization phenomenon within the lower female genital tract, in which anatomically related epithelial surfaces are exposed to shared carcinogenic influences and become susceptible to multifocal neoplastic transformation. This model is particularly attractive when considering synchronous HPV-related lesions affecting multiple sites. A second possibility is divergent clonal evolution, in which lesions arising in different epithelial compartments, or even within a single lesional field, acquire distinct secondary molecular alterations over time. In the present case, the cervical lesion retained a conventional HPV-associated immunophenotype, whereas the vulvar lesion showed an unusual combined p16/p53 profile, which may reflect either divergent molecular evolution of an initially HPV-related process or the coexistence of biologically distinct epithelial clones. In the absence of HPV-specific and *TP53* molecular studies, these possibilities remain speculative but are important conceptual frameworks for interpreting the case.

### 3.4. Diagnostic Implications and Study Limitations

From a diagnostic perspective, the present case highlights the importance of interpreting immunohistochemical findings within the broader morphological and clinical context. While p16 and p53 immunohistochemistry are widely used as adjunctive tools in the evaluation of vulvar precursor lesions, unusual staining combinations may occasionally occur and should not be overinterpreted as proof of a specific molecular pathway in the absence of confirmatory testing. In the present case, the diagnosis of vulvar HSIL was favored primarily on morphologic grounds, with immunohistochemistry serving as an adjunctive rather than definitive etiopathogenetic tool [6]. One strength of the present report is the clear juxtaposition of two synchronous lower genital tract squamous intraepithelial lesions with different immunophenotypic profiles in the same patient. This internal comparison makes the case educational from a diagnostic perspective, because the cervical lesion showed a conventional pattern while the vulvar lesion illustrated an interpretative pitfall. In addition, the case provides a morphologic–immunohistochemical correlation that may be useful for pathologists encountering vulvar lesions that do not fit neatly into the conventional binary model. On the other hand, several limitations of this report should be emphasized. Most importantly, direct HPV testing was not performed for either lesion, and molecular analysis of *TP53* was not available for the vulvar lesion. Direct HPV testing was not performed because of logistical and resource limitations at the time of case evaluation. Therefore, although the cervical lesion showed a conventional HPV-associated immunophenotype and the vulvar lesion demonstrated an unusual p16 block-type/p53 overexpression profile, the etiopathogenetic interpretation remains inferential. Immunohistochemistry alone cannot definitively establish transcriptionally active HPV infection or confirm the presence and type of *TP53* mutation. Accordingly, this case should be interpreted as a morphologically and immunophenotypically unusual presentation rather than as molecular proof of pathway overlap.

Similarly, HPV status was inferred indirectly based on the block-type p16 immunohistochemical expression observed in the cervical lesion. Although p16 overexpression is widely accepted as a surrogate marker of transcriptionally active high-risk HPV infection, direct molecular confirmation through HPV genotyping or viral DNA detection was not performed. Consequently, the precise molecular mechanisms underlying the coexistence of the cervical and vulvar lesions cannot be fully elucidated in the absence of confirmatory molecular analyses.

Future studies incorporating comprehensive molecular profiling, including next-generation sequencing, may help clarify the biological mechanisms responsible for mixed immunophenotypic patterns in vulvar intraepithelial lesions. At the same time, continued reporting of rare gynecologic neoplasms and unusual lower genital tract precursor lesions remains important, as carefully documented individual cases may highlight diagnostic pitfalls, broaden the recognized spectrum of vulvar and cervical squamous neoplasia, and provide a basis for improved clinicopathologic interpretation in future similar cases [25,26,27,28]. In particular, cases with unusual or discordant immunophenotypes may help refine current interpretative criteria, especially when morphology and immunohistochemistry do not align in a straightforward manner. While single-case observations cannot resolve these questions definitively, they remain valuable because they can stimulate further molecular investigations, improve diagnostic awareness, and support more thoughtful clinical and therapeutic stratification in comparable presentations [6,9,16].

## 4. Conclusions

In summary, this case illustrates synchronous cervical and vulvar high-grade squamous intraepithelial lesions with divergent immunophenotypic profiles, including a vulvar lesion showing an unusual combined p16 block-type/p53 overexpression pattern. Beyond its rarity, the case highlights a practical diagnostic challenge: vulvar precursor lesions with discordant morphology and immunohistochemistry should not be classified on the basis of a single biomarker alone. Instead, accurate interpretation requires integration of morphology, immunohistochemistry, clinical context, and, where available, molecular testing. In the absence of direct HPV testing and TP53 molecular analysis, the etiopathogenetic significance of this combined immunophenotype remains hypothetical.

## Figures and Tables

**Table 1:** Diagnostic and clinical comparison of conventional vulvar HSIL, differentiated VIN, and the present case.

Feature	Conventional Vulvar HSIL	Differentiated VIN (dVIN)	Present Case
Usual etiologic association	High-risk HPV-related	HPV-independent	Etiology uncertain; morphology favored HSIL, but immunophenotype was unusual
Typical patient profile	More often younger women	More often older women	83-year-old woman
Morphology	Full-thickness atypia, loss of maturation, mitoses above basal layer	Basal/parabasal atypia, premature maturation, dyskeratosis, keratinization	Full-thickness atypia and loss of maturation favored HSIL; no prominent keratinization, dyskeratosis, or premature maturation identified
p16 staining	Block-type positive	Usually negative or non-block-type/focal	Block-type positive
p53 staining	Usually wild-type	Often aberrant	Aberrant overexpression
Main diagnostic pitfall	Usually limited	May overlap with other atypical vulvar lesions	Mixed p16/p53 profile may suggest conflicting pathways
Clinical implication	Managed as HPV-associated precursor lesion	Greater concern for HPV-independent pathway and progression risk	Requires cautious interpretation and clinicopathologic correlation
Molecular confirmation needed?	Not always, but may be useful in difficult cases	Particularly useful in difficult cases	Unavailable at the time of publication

## Data Availability

The original contributions presented in this study are included in the article. Further inquiries can be directed to the corresponding author.
